# Measurement, Validation and Uncertainty of an Experimental Procedure to Characterize the Size-of-Source Effect of Radiation Thermometers, in the Framework of an Industrial Calibration Laboratory

**DOI:** 10.3390/s22218284

**Published:** 2022-10-28

**Authors:** Javier de Lucas

**Affiliations:** Instituto Nacional de Técnica Aeroespacial (INTA), Centro de Metrología y Calibración, Ctra. de Ajalvir, km 4, 28850 Torrejón de Ardoz, Spain; delucasvj@inta.es

**Keywords:** radiation thermometer, thermometry, industrial calibration, metrology, traceability, blackbody, emissivity

## Abstract

An experimental procedure for characterizing the size-of-source effect (SSE) is proposed. Such an effect is the cause of one of the main influence variables generating uncertainty in the measurement, both in calibration and use, of direct reading radiation thermometers (RT). The procedure and uncertainty calculation described in the paper are aligned in terms of metrological traceability, with the requirements generally imposed to ensure the accuracy of measurements in industry and science. Results of application and validation of this particular procedure with equipment, including black body (BB) sources normally used in radiation thermometry calibration laboratories in the industrial field, are shown.

## 1. Introduction

Radiation thermometry and qualitative or quantitative thermography (thermal imaging) mainly between −50 °C and 500 °C have experienced a strong impulse in the last decades. The newest technologies use low-cost, uncooled micro-bolometers in the FPA (focal plane array) or thermopiles. In the past, bolometers needed cryogenic cooling, usually by a miniature Stirling cycle refrigerator or liquid nitrogen. In addition, the improvement of FPA resolution has allowed up to 1280 × 1024 pixels, extremely high for current thermography systems, which in general have limited resolution due to the size required for single detectors to integrate thermal radiation. Bolometers and thermopiles are thermal sensors that measure object temperature as a function of changes in the sensor temperature itself.

Some of the most interesting applications of non-contact thermometry include food industry, building maintenance and inspection, energy management, air conditioning, performance of electric and electronic circuits, non-destructive thermal testing in aeronautics and space, security and defense, etc. The equipment generally used to measure temperature and/or to show a thermal image, operate in the bands (3 to 5) μm (MWIR or Medium Wavelength Infrared) and (8 to 14) μm (LWIR or Long Wavelength Infrared). In general, optimum spectral band choices for infrared imaging applications, can require the use of LWIR for scenes with a very wide temperature range with the presence of both hot and cold objects. Atmospheric absorption, object temperature, presence of smoke or moisture in the atmosphere, etc., are factors that can directly affect the choice of spectral band.

Having reliable measurements requires ensuring metrological traceability to the International System of Units (SI), [1,2]. All the calibrations constituting the chain that links the current measurement with the practical realization of the unit (kelvin in temperature) contribute to the final uncertainty. In general, calibration laboratories are accredited by accreditation bodies members of ILAC (International Laboratory Accreditation Cooperation) (https://ilac.org/ (accessed on 1 October 2022)), under the standard ISO/IEC 17,025 [3], some of which in Europe are: ENAC (https://www.enac.es/ (accessed on 1 October 2022)) in Spain, DAkkS (https://www.dakks.de/en/home-en.html (accessed on 1 October 2022)) in Germany, UKAS (https://www.ukas.com/ (accessed on 1 October 2022)) in United Kingdom, COFRAC (https://www.cofrac.fr/ (accessed on 1 October 2022)) in France, ACCREDIA (https://www.accredia.it/ (accessed on 1 October 2022)) in Italy, SAS (https://www.sas.admin.ch/sas/en/home.html (accessed on 1 October 2022)) in Switzerland, etc. Accredited calibration laboratories act as a link between SI and end users, namely industry, market, R&D centers, other calibration laboratories, etc. Currently in Spain, for example, there are 21 laboratories accredited by ENAC to calibrate radiation thermometers, thermal imagers and black body sources.

One of the most important influence variables that contributes to the uncertainty in non-contact temperature measurement using RT is the size-of-source effect (SSE). This means that radiation coming from points outside the theoretical target of the optical system can reach the detector due to optical imperfections (aberrations, internal reflections, diffraction, etc.). Similarly, radiation within the field of view (FOV) may not reach the detector. SSE is usually evaluated (direct method) by measuring RT signal focusing the optics on a uniform radiation source, usually a high effective emissivity (*ε_a_*) BB (cavity or extended area) source, with interchangeable circular apertures of different diameter. Alternatively (indirect method) SSE can be evaluated by measuring the radiation of the region outside the target, which is in turn blocked with a small (ideally black) disk. However, the indirect method is difficult to automate and requires more experimental effort [4], thus the direct method is generally chosen by industrial calibration laboratories.

RTs calibrated using sources of certain aperture or diameter size cannot be used (without making corrections) on targets of different sizes. On the other hand, metrological characterization of BB (reference radiation sources for RT and TI calibration) is usually carried out with a standard BB and a RT acting as a comparator. A necessary condition to correctly transfer from the standard BB to the source to be calibrated is to know how to correct RT readings when sources have different diameters or even when temperature gradient around the apertures differs. As we will see, the corrections are linear with wavelength, being greater in general, in low-cost commercial RT (not too sophisticated optics) in the LWIR band.

In general, industrial calibration laboratories (accredited or not), in the field of radiation thermometry, provide calibration results only for the aperture size of their own BBs used as reference standards. Although the calibrations are correct for the strict measurement conditions indicated in the calibration certificates, they are not sufficient or have limited utility if, as usual, the end user utilizes the RT (in his laboratory or in an industrial environment), focusing it on sources of different size.

This paper presents an experimental procedure based on the direct method, simple and easy to implement in the framework of industrial calibration laboratories. The particular procedure is aligned with a mathematically simple approximation of the general SSE model. It is based on the use of a well-characterized BB and a system or set of external elements delimiting the apertures, which can be cooled to minimize the effect of background radiation. Uncertainty calculation of the basic magnitude characterizing SSE, which serves to calculate a correction on the RT temperature reading between different source diameters, is exposed in some detail. Finally, the procedure is illustrated, applying it to commercial equipment, and is validated with a BB (different from that used to SSE characterization) working at various temperatures and source sizes. The application of the procedure, adapted to the particularities of each laboratory in terms of availability of equipment, methodology for data acquisition, type of thermometers for contact temperature measurements, etc., would allow significant improvements in the information contained in the calibration certificates, making such information more useful to end users.

## 2. Materials and Methods

### 2.1. Basic Model for Calculation of SSE Corrections in RT Calibration and Use

In general, a direct-reading RT generates in its detector a signal *S* which is linear with the radiance of the source. Assuming absolute temperature *T* in kelvin and emissivity (*ε*) equal to 1 (blackbody source), in absence of SSE the signal is:

(1) $$S\left(T\right)=g\overset{\infty }{\underset{0}{\int }}s\left(\lambda \right)L_{b}\left(\lambda ,T\right)d\lambda ≅\frac{C}{e^{\frac{c_{2}}{AT+B}}-1}\equiv S_{sh}\left(T\right)$$

In the second term of (1), *s*(*λ*) is the relative spectral responsivity of the thermometer, *g* is a constant dependent on the geometrical, optical and electrical properties of the thermometer and *L_b_*(*λ*, *T*) is the spectral radiance of a blackbody at wavelength *λ* and temperature *T*, given by Planck’s law.

The integral in (1) is well approximated by the Sakuma–Hattori (*S_sh_*) interpolation equation [5], which includes the effect of bandwidth, *c*_2_ is the second radiation constant 0.0014388 m·K and *C* is a proportionality constant. Being *λ_x_* the so called temperature-dependent extended effective wavelength, for most radiation thermometers it is well approximated by a linear function of inverse temperature: *λ_x_* = *A* + *B*/*T*, *A* and *B* being specific parameters of each RT, depending on its spectral responsivity, i.e., detector, filter and lens transmittance, waveband, etc.

If *d_t_* is the RT nominal target size (diameter) at a specified distance, during calibration it is normally used a source with uniform temperature *T* and aperture diameter *d* > *d_t_*. If the source temperature is high enough (in general it is considered *t* > 200 °C) the background radiation effect, at typical laboratory temperature, *t_w_* ≅ 23 °C, can be assumed negligible [4,6] and the measured signal *S_T,d_* reduces (with *T* and *d* as variables of interest) to:

(2) $$S_{T,d}=\xi \left(d\right)S_{sh}\left(T\right)$$

The proportionality factor *ξ*(*d*) is independent of *T* and contains all the information concerning the RT optical defects that give rise to SSE. Then, *S_sh_* can be considered the ideal signal-temperature relationship for the RT, i.e., in the absence of SSE or for an infinite diameter source.

In this case, the quantity describing the effect in the direct method, is defined by:

(3) $$\sigma \left(d\right)=\frac{S_{T,\;d}}{S_{T,\infty }}=\frac{\xi \left(d\right)}{\xi \left(\infty \right)}$$

Using (3), *σ*(*d*) − *σ*(*d_t_*) represents the ratio between the signal from the outer ring to the target corresponding to a source of diameter *d* and the maximum signal corresponding to a source of infinite diameter. Usually, industrial RT manufacturers assume, for the definition of nominal target size, signal levels of 90% (*d*_90_) or 95% (*d*_95_) of the maximum. However, (though less common) they also specify the target size, as that corresponding to the optical field of view (FOV), i.e., the size of the image of the field stop (normally the detector) by the optical system [7]. It has been seen that, for direct reading RT of the type frequently used in industrial applications, for source diameter (typically the maximum diameter d_0_ of a BB cavity source without external aperture plates), *d*_0_ ≥ 5*d*_90_, RT signal achieves the maximum value and remains constant, i.e., *S*(*d* ≥ *d*_0_) ≅ *S*(∞) [8].

In calibration laboratories, SSE is usually measured using large aperture cavity BB sources, with cylindrical or cylinder-conical geometry, always with high (≅1) and uniform (at the bottom cavity at least) effective emissivity *ε_a_*. Additionally, BB temperatures are high enough in relation with that of the external plates or elements delimiting the variable apertures. These plates or machined pieces form a set with diameters *d_t_* ≤ *d* ≤ *d*_0_ and are mounted at the front of the source.

Characterizing SSE is a complementary task to the complete calibration work of RT. The results of a calibration are usually given in terms of a fixed diameter source *d_c_*. In order to facilitate the calculation of corrections to the RT reading, between the calibration diameter *d_c_* and other different *d*, (3) is used to define the quantity:

(4) $$\sigma^{*}\left(d,d_{c}\right)=\sigma \left(d\right)-\sigma \left(d_{c}\right)=\frac{S_{T,d}-S_{T,d_{c}}}{S_{T,d_{0}}}$$

In first approximation, it can be assumed *σ*(*d*) ≅ 1 and, with (4), it is easy to see that:

(5) $$\xi \left(d\right)≅\xi \left(d_{c}\right)\left(1+\sigma^{*}\left(d,d_{c}\right)\right)$$

For an input signal *S*, direct reading RT shows a temperature value *I* (display indication), implicitly dependent on temperature and diameter (area in general) of the source, *I* (*T*, *d*). Typically, this kind of RT is provided with an emissivity function *ε_instr_* selectable between 0 and 1. This parameter is called instrumental emissivity adjustment and facilitate the use of RT to measure temperature of surfaces different to perfect black bodies. In general, *I* also depends on detector temperature *T_d_* and ambient temperature *T_w_* [9]. In normal laboratory ambient conditions, it can be assumed *T_d_* ≅ *T_w_* when RT is not too close to the BB aperture at high temperatures. Provided that there are no additional correction factors aside from SSE, *I* can be obtained from:

(6) $$S_{sh}\left(I\left(T,d\right)\right)=\xi \left(d\right)\frac{\varepsilon_{a}}{\varepsilon_{instr}}S_{sh}\left(T\right)+\frac{\left(\varepsilon_{instr}-\varepsilon_{a}\right)}{\varepsilon_{instr}}S_{sh}\left(T_{w}\right)$$

Both *T* and *d* are considered the main variables, while *ε_a_*, *ε_instr_* and *T_w_* are fixed parameters. Typically, laboratory conditions allow simplify (6), by selecting *ε_instr_* = 1, and by the use of a cavity BB with *ε_a_* ≅ 1 at a temperature *T* much larger than *T_w_*. Then, the second summand in (6) can be neglected and the correction Δ*I*, when RT is used to measure the temperature of a source with diameter different from that used in calibration *d_c_*, can be obtained calculating *S_sh_*(*I*(*T*, *d_c_* + Δ*d*))/*S_sh_*(*I*(*T*, *d_c_*)). Considering, by definition, *I*(*T*, *d_c_* + Δ*d*) = *I* + Δ*I* and *d* = *d_c_* + Δ*d*, by developing at the first order, we obtain:

(7) $$\Delta I=\sigma^{*}\left(d,d_{c}\right)\frac{S_{sh}\left(I\right)}{\left(dS_{sh}/dT\right)\left(I\right)}$$

With (1), we can finally calculate the correction to RT temperature reading *I*, as a function of source diameters *d*:

(8) $$C_{SSE}=\Delta I=\sigma^{*}\left(d,d_{c}\right)\frac{\lambda_{x}I^{2}}{c_{2}}\left(1-e^{\frac{-c_{2}}{\lambda_{x}I}}\right)$$

**Figure 1:**
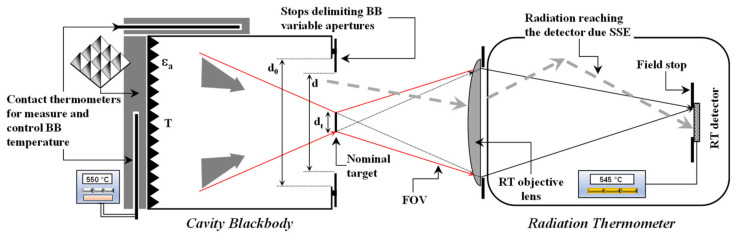
Basic configuration for SSE measurement of a RT, with a BB cavity source of variable aperture. Dashed gray arrows represent rays reaching the RT detector due SSE. Solid red and black arrows represent rays reaching the detector, within the theoretical field of view (FOV).

Figure 1 shows schematically the basic principles of SSE measurement.

In (8), *λ_x_* depends on RT spectral responsivity (optics, filters and detector). Its value can be calculated by means of direct calibration of RT against reference BB, using (1) for fitting the parameters *A*, *B* and *C*, provided that RT internal signal, linear with the radiance, may be accessible [10,11]. Nevertheless, in industrial direct-reading RT this is not possible in general. For SSE characterization and uncertainty evaluation of this type of instruments, it is still possible to assume rectangular spectral response in the waveband Δ*λ* = (*λ*_2_ − *λ*_1_) [12]. Then, it can be proved that *A* = *λ*_0_(1 − 6(*ζ*/*λ*_0_)^2^) and *B* = (*c*_2_/2)(*ζ*/*λ*_0_)^2^, being *λ*_0_ = (*λ*_1_ + *λ*_2_)/2 and *ζ* = Δ*λ*/√12.

For non-uniform radiation sources, the mathematical model for calculating SSE corrections is quite complex and difficult to solve in general. If the element delimiting aperture (as in Figure 1) is very close to BB, cooling the stops could not be possible and, depending on RT, radiation emitted by these elements, may not be negligible. The set formed with the BB and the element defining aperture defines an effective (or apparent) diameter *d_eff_* considered as that of a uniform source producing the same signal in the RT [13]. The value *d_eff_* depends on the particular RT and also on the temperature gradients existing in the piece defining the aperture, including the front plate of the source.

### 2.2. Description of the Experimental Facility

The basic facility used in this research consists of a high effective emissivity BB cavity source, a set of brass machined apertures of different diameters placed in front of the cavity entrance and a system for cooling these pieces. The use of extended area or flat plate BB sources are not recommended for this application. This is due to its worse uniformity and low emissivity. In addition this quantity can also vary over time due to multiple reflections between the BB surface and the elements used to generate the variable apertures. Then the value *ε_a_* of that type of systems can depend on aperture diameter. This is negative, because the observed variations in RT readings can be due ether to SSE or *ε_a_*. For cavity BBs with high length to diameter ratios, *ε_a_* is near 1 at the bottom and can be numerically calculated. In this sense, it is very important to assure that radiation reaching RT comes only from the cavity bottom, where the source is more (spatially) uniform in radiance and where surface temperature is better defined and can be easily measured with contact probes. Bottom or surface temperature has to be continuously recorded in order to correct RT readings for variations due to a greater leak heat in form of convection when BB aperture increases [14]. The RT is placed in front of the aperture at a fixed distance, with its optical axis coinciding with that of the cylinder-cone and always searching for the maximum signal. Target size *d_t_* depends on the distance and it is usually specified by the manufacturer.

#### 2.2.1. Reference BB

**Figure 2:**
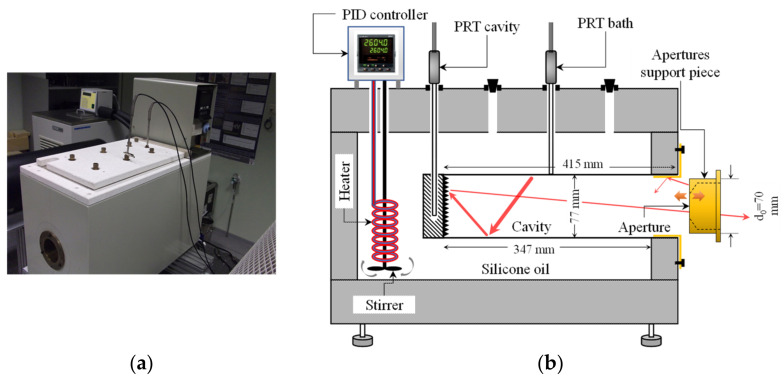
(**a**) BB used in this research as reference radiation source for SSE characterization; (**b**) Detailed scheme of the system, showing the bath, reference thermometers, cavity and support piece for the apertures. Red arrows represent rays emitted and reflected by the BB internal surfaces.

The BB used in this experimental procedure is a source designed and manufactured by National Physical Laboratory NPL, UK, for INTA in 1993, based on a commercial GRANT calibration bath, provided with an oxidized stainless steel cylindrical cavity, with surface intrinsic emissivity *ε* = 0.85, for the spectral band between 0.9 μm and 14 μm. With length 347 mm and diameter 77 mm, the bottom cavity is grooved (as seen in Figure 1) in order to increase internal reflections and therefore *ε_a_*. The cavity is immersed in a silicone oil isothermal bath with a PID temperature controller, enabling stability and uniformity better than ±0.05 °C around the cavity, in the temperature range between 30 °C and 180 °C. Figure 2 shows an opening at the front of the tank, where the cavity is attached, allowing radiation towards the exterior.

BB and bath temperatures are measured with Platinum Resistance Thermometers (PRT) of the type Pt100 (100 Ω nominal at 0 °C). The PRT that measures BB temperature is placed in a thermo-well drilled at the rear of the cavity, as shown in Figure 2b. The other PRT is immersed in the bath for checking of temperature uniformity. The metrological facility is completed with a thermometric resistance bridge ASL model F700 and a 100 Ω standard resistor TINSLEY model 5685A, at controlled temperature of 36 °C in air. PRTs, resistance bridge and standard resistor are periodically calibrated, with metrological traceability to SI, in accredited calibration laboratories that use the standard ISO/IEC 17025 [3], ensuring that each contribution to the final uncertainty in SSE characterization is completely quantified.

For computing *ε_a_* uniformity at the cavity bottom, we use a numerical model based on backward ray-tracing and Monte Carlo method [15] for non-isothermal cavities. For a given fixed geometry and intrinsic emissivity *ε* (together with its uncertainty), *ε_a_* only depends on the cavity longitudinal temperature gradient and aperture diameter. In order to prevent excessive heating of the aperture supporting piece (Figure 2b), thus allowing its external cooling, it has been moved towards the outside, focusing the RT on the aperture perpendicular plane, as shown in Figure 1. Such configuration does not affect *ε_a_*, because the radiation from the piece towards the cavity bottom can be considered negligible compared with that from the cavity at much higher temperature. On the other hand, we have experimentally checked that, with the piece at that position, heat leaks due to convection (and therefore cavity temperature) are less dependent on the aperture size. With this set-up, the resulting cavity has 415 mm length, inner diameter 77 mm and maximum aperture diameter 70 mm.

The cavity longitudinal temperature gradient has been measured by placing the secondary PRT in several holes drilled through the bath upper cover (Figure 2a). On the other hand, for measurement of the temperature gradient at the inner surface of the brass piece connecting the cavity with the exterior, we have used a thermal imager. This piece is the yellow one which is attached to the bath in Figure 2b.

**Figure 3:**
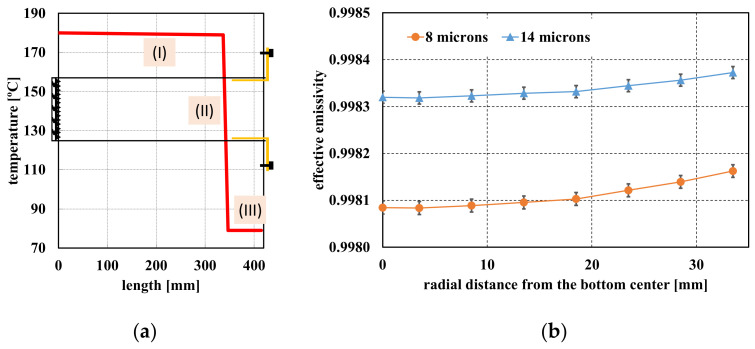
(**a**) Longitudinal temperature gradient measured at the BB cavity; (**b**) Radial profiles for the effective emissivity in 8 μm and 14 μm, as a function of the distance from the center.

For bottom temperature of 180 °C, gradient was measured and subsequently approximated by a profile with three linear segments: (I) 1 °C decreasing up to 337 mm from the bottom, (II) 100 °C decreasing up to the beginning of the brass piece and (III) uniform, with average temperature of 79 °C, along the 68 mm of that piece. The profile is shown in Figure 3a.

For non-isothermal cavity, *ε_a_* has spectral dependence, therefore we have calculated its value for the spectral band between 8 μm and 14 μm. The results are shown in Figure 3b. The error bars represent the standard deviation proper of the simulation model and the Monte Carlo method, (*ε_a_*(1 − *ε_a_*)/N)^1/2^ ≅ 1.4 · 10^−5^, N = 10^7^ being the number of trials computed.

The estimation of *ε_a_* and thus the radial uniformity of the BB radiance temperature, was carried out averaging the values showed in Figure 3b. The maximum variation was calculated as half of the difference between the maximum at 14 μm and the minimum at 8 μm. The results were *ε_a_* ± Δ*ε_a_* = 0.9982 ± 1.4 · 10^−4^, Δ*ε_a_*, in terms of radiance temperature, represents 0.02 °C at 8 μm and 0.03 °C at 14 μm, when BB temperature is 180 °C. Taking into account the expected uncertainties in the SSE corrections, the estimated temperature radiance uniformity through the 70 mm aperture is considered very acceptable.

#### 2.2.2. System of Interchangeable Apertures

**Figure 4:**
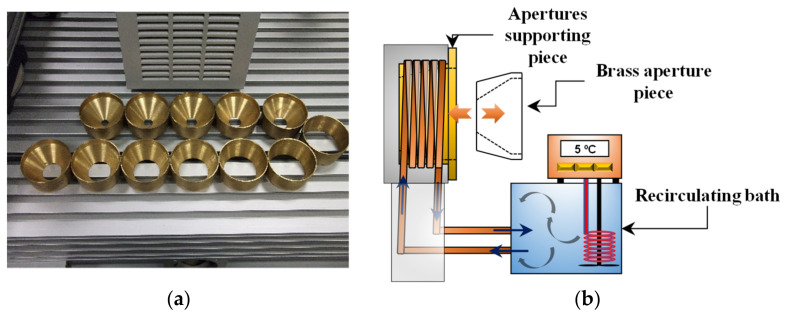

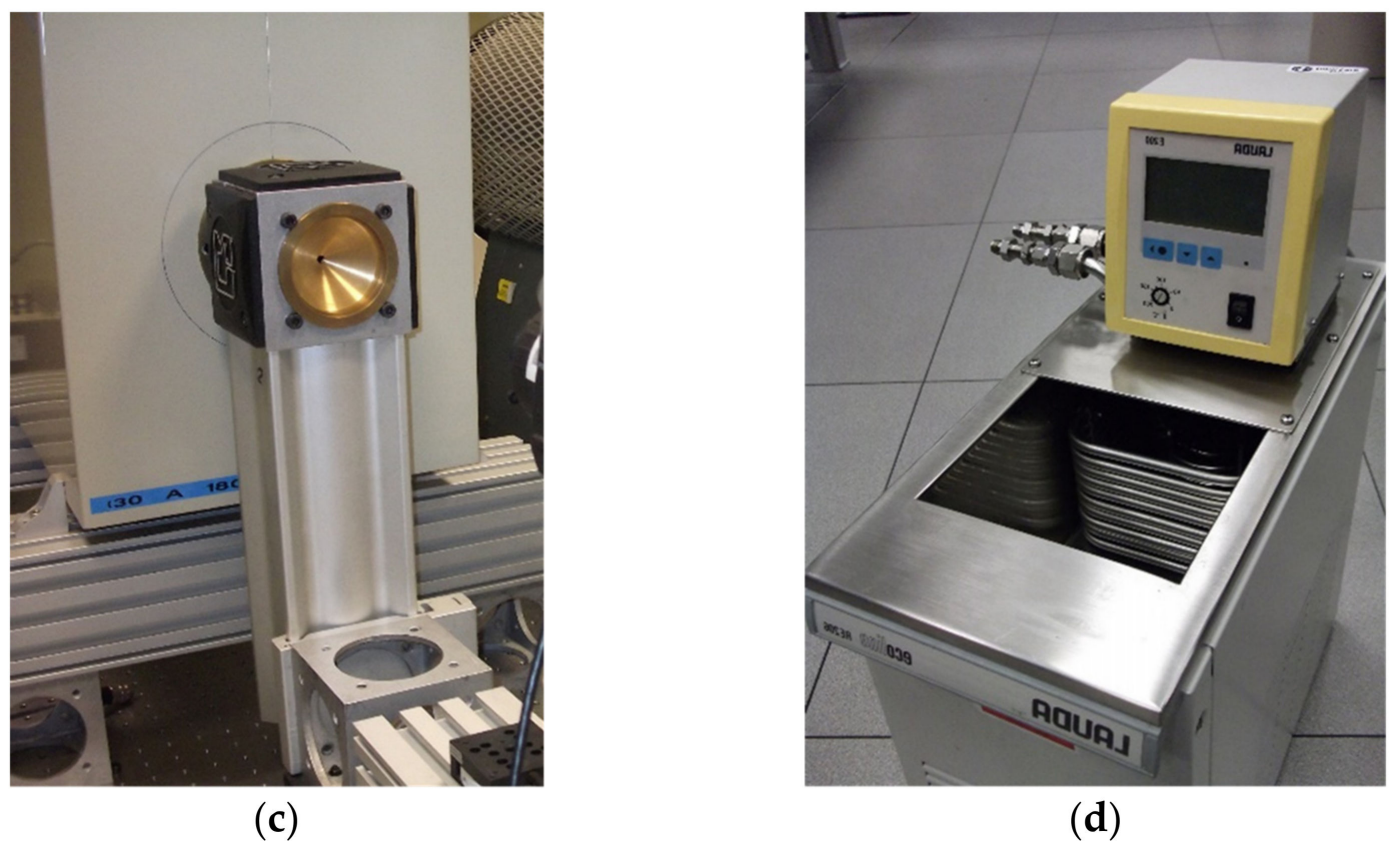
(**a**) Interchangeable brass pieces defining the variable apertures; (**b**) Scheme of the apertures cooling system. Orange arrows indicate the fitting of the brass pieces on the structure; (**c**) Structure for positioning the apertures in front of BB; (**d**) Cooling recirculating bath.

In order to modify the size of source, a set of 12 interchangeable apertures (Figure 4a) were designed and machined. They are made of brass and have diameters between 5 mm and 60 mm. These can be easily inserted in the supporting piece, as shown in Figure 4b, which can be in turn cooled by means of a copper coil wound around the piece, in good thermal contact with it. The coil is then connected to a recirculating bath model LAUDA, E200 Ecoline RE 204 (Figure 4d) and, in turn, the supporting piece is mounted in a structure designed to allow its positioning in front of the BB entrance (Figure 4c).

With the system described, the apertures can be cooled up to 5 °C. It has been observed that the time required to stabilize the temperature of the pieces defining apertures, after each operation of piece changing is about 10 min. Then, to avoid errors due to a possible drift of BB temperature (the effect of BB stability), continuous recording with the cavity PRT is needed. The RT readings for each aperture, have to be corrected and standardized to the temperature corresponding to the first aperture. We will come back to this in the next section.

### 2.3. Metrological Traceability

The reference standards: ratio resistance bridge (with its associated reference resistor) and PRTs of BB are periodically calibrated and the corresponding uncertainty *U* (expanded for a coverage factor *k* = 2 or coverage probability of 95%) is calculated and expressed following the recommendations of the BIMP GUM guide [16]. The metrological traceability is then assured and documented through calibration certificates emitted in the framework of the Spanish national accreditation body (ENAC) (schedule of accreditation nº No.16/LC10.007 [17]), or National Metrology Institutes (NMI). The RPTs are calibrated by the method of comparison between 0 °C and 200 °C, with maximum expanded uncertainty of 0.02 °C. The bridge is calibrated in ratio values between 0 and 4, with expanded uncertainty of 8·10^−7^, whereas the uncertainty of the resistance of the standard resistor (100 Ω nominal) is 1.4 ppm (*k* = 2).

### 2.4. Measurement Process, Model for Analysis of the Results and Uncertainty Calculation

Once the source and aperture temperatures are stabilized, a representative number of RT readings *I* is taken in order to obtain average and standard deviation (measure of dispersion or repeatability). The reproducibility of the procedure is evaluated by taking a second series of measurements, refocusing and realigning the RT on the aperture of minor diameter (*d*_1_), always searching for the maximum signal. It is advisable to select *d*_1_ large enough (and of course > *d_t_*) because small errors in target centering can lead to dispersion in the results and thus, to an increasing of uncertainty in SSE characterization for small sources.

With the pairs obtained experimentally {*d_i_*, *I_i_*}, being *d_t_* < *d_i_* ≤ *d*_0_, we calculate σ_i_*(*d_i_*, *d_c_*) with (1) and (4), being *S_T,d_* = *S_sh_*(*I*), defining *C* = 1, and with *λ_x_*(*A*, *B*) function of the RT bandwidth. The corresponding corrections between arbitrary diameters *d_y_* and *d_z_* are calculated with σ*(*d_y_*, *d_z_*) = σ*(*d_y_*, *d_c_*) − σ*(*d_z_*, *d_c_*). For data fitting, we have proposed a reference function *σ_SSE_*(*d*), that represents adequately the general behavior observed in most commercial direct-reading RT. The fitting function must fulfil *σ_SSE_*(*d_c_*) = 0 and *σ_SSE_*(*d*) → constant, when *d* → ∞. Taking this in mind, we have opted by a function dependent of three free parameters (*a*, *b*_1_, *b*_2_):

(9) $$\sigma_{SSE}\left(d\right)=a(1-e^{b_{1}\left(d_{c}-d\right)+b_{2}{\left(d_{c}-d\right)}^{2}})$$

If, as it is expected, *d*_0_ is large enough, in general it holds *a* ≅ σ*(*d*_0_, *d_c_*) and the calculation of *b*_1_ and *b*_2_ reduces to a least squares fitting of the set {*d_i_*, log(1 − σ_i_*/*a*)} to the function (linear in the parameters): *b*_1_(*d_c_* − *d*) + *b*_2_(*d_c_* − *d*)^2^.

The uncertainty calculation of the RT reading *I*, due to all the significant influence quantities or variables affecting the temperature measurement with this kind of instruments [7], is a complex task that exceeds the general objectives and aims of this paper. In this work, we only consider the uncertainty *u*(σ*) and how it propagates to the uncertainty of the correction Δ*I*, that has to be applied to the indication *I*. As usual, we follow the methodology given in GUM guide [16], for calculation, propagation and expression of standard u and expanded *U* = 2*u* (or for *k* = 2) uncertainty.

The measurement model function for σ* is given in Equation (4). It depends on five influence quantities {*x_k_*}*_k_*_=1,…,5_: diameters *d* and *d_c_*, RT reading *I* for source diameter *d*, reading *I_c_* for the calibration diameter *d_c_* and *I*_0_ for the maximum diameter *d*_0_. The mathematical model bases on the validity of (5) independently of *d*_0_, so this quantity is not an influence variable for the uncertainty. The five characteristic variables of the model are independent (no correlation or negligible) and therefore, the standard uncertainty can be written as:

(10) $$u\left(\sigma^{*}\right)=\sqrt{\overset{5}{\underset{k=1}{\sum }}{\left(\frac{\partial \sigma^{*}}{\partial x_{k}}\right)}^{2}u^{2}\left(x_{k}\right)}$$

With some algebra, it can be seen that the contribution of readings is:

(11) $$\begin{array}{c} {\left(\frac{\partial \sigma^{*}}{\partial I}\right)}^{2}u^{2}\left(I\right)+{\left(\frac{\partial \sigma^{*}}{\partial I_{c}}\right)}^{2}u^{2}\left(I_{c}\right)+{\left(\frac{\partial \sigma^{*}}{\partial I_{0}}\right)}^{2}u^{2}\left(I_{0}\right)\\ \;\;\;\;\;\;={\left(\left(1+S_{sh}\left(I_{0}\right)\right)\frac{c_{2}}{\lambda_{x}I_{0}{}^{2}}\right)}^{2}\left(u^{2}\left(I\right)+u^{2}\left(I_{c}\right)+\sigma^{*}{}^{2}u^{2}\left(I_{0}\right)\right) \end{array}$$

and the contribution of diameters, using (9) is:

(12) $$\begin{array}{c} {\left(\frac{\partial \sigma^{*}}{\partial d}\right)}^{2}u^{2}\left(d\right)+{\left(\frac{\partial \sigma^{*}}{\partial d_{c}}\right)}^{2}u^{2}\left(d_{c}\right)\\ \;\;\;\;\;\;={\left(\left(\sigma^{*}-a\right)\left(b_{1}+2b_{2}\left(d_{c}-d\right)\right)\right)}^{2}\left(u^{2}\left(d\right)+u^{2}\left(d_{c}\right)\right) \end{array}$$

As standard uncertainty of each RT reading, we have taken the maximum value among the resolution (usually the numerical temperature value showed in the display) and the short-term temperature stability of the BB (given by the PRTs). In the case of resolution, we consider a type B estimation of uncertainty, as recommended in GUM [16], assuming rectangular probability distribution function, taking half of the last significant digit (*r*) divided by √3. For the stability we use, as usual, the experimental standard deviation of the mean (ESDM, *s*). If the BB temperature drifts (long term stability), the drift is corrected standardizing the current reading *I_i_* with the difference with respect to the one obtained using the first aperture, i.e., *I_i_* → *I_i_* + (*t*_1_ − *t_i_*).

It is defined *u*_1_(*I_i_*) = max{*r*/√12; *s_i_*/√n}, being *s_i_* the ESDM for *n* readings taken with diameter *d_i_*. The influence in the uncertainty of the approximation (5), is quantified with a contribution obtained from the difference between the correction measured *C_i_^med^* = (*I_i_* − *I_c_*) and the correction calculated by means of (8). The contribution, written as a standard uncertainty component, is defined by *u*_2_(*I_i_*) = │*C_i_^med^* − *C_i_^cal^*│/√3. The reproducibility of the procedure is also taken into account. We consider the difference between two measurements series, the second one corresponding to a new RT realigning and refocusing, as was explained in Section 2.4. Thus, the contribution is defined by *u*_3_(*I_i_*) = │*I_i_^series^*^1^ − *I_i_^series^*^2^│/√12, where explicitly we assume that the current indication *I_i_* is the average between that of the two series. Combining the three components, we have finally:

(13) $$u^{2}\left(I_{i}\right)={\left(\max\{\frac{r}{\sqrt{12}};\frac{s_{i}}{\sqrt{n}}\}\right)}^{2}+{\left(\frac{\left|C_{i}^{med}-C_{i}^{cal}\right|}{\sqrt{3}}\right)}^{2}+{\left(\frac{\left|I_{i}^{series1}-I_{i}^{series2})\right|}{\sqrt{12}}\right)}^{2}$$

The uncertainty of the correction between two arbitrary diameters (within the range between *d*_1_ and *d*_0_) is calculated with (8). For that purpose, we consider it necessary to add another uncertainty factor to (10) *u_extra_*, which quantifies the quality of the fit. It is common, being conservative, to take the maximum residual considered as the absolute limit of a rectangular distribution function, i.e., *u_extra_* = max{│σ_i_* − *σ_SSE_*(*d_i_*)│}/√3.

## 3. Results

**Figure 5:**
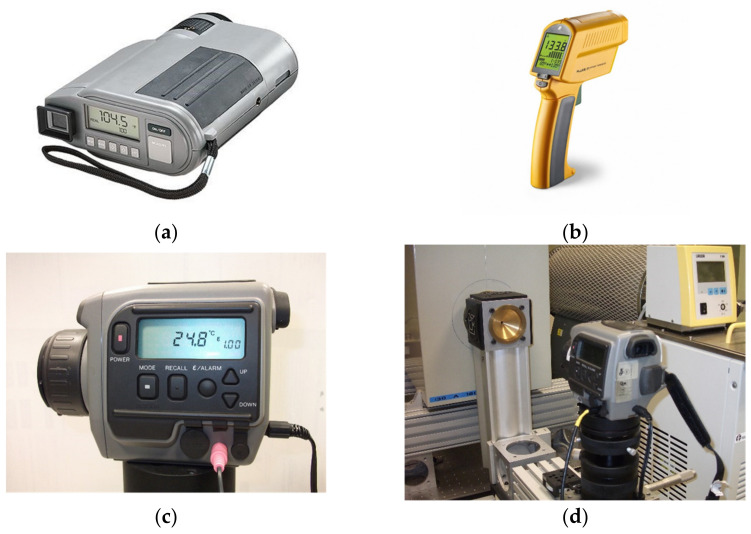
Commercial direct reading RTs used in this research. (**a**) Model IRCON UX40P; (**b**) Model FLUKE 574CF; (**c**) Model LAND C300; (**d**) C300 during measurements.

The procedure described in this work has been applied to typical direct-reading RT used commonly in industrial applications, either in a laboratory environment or in industrial facilities. In general, these thermometers are equipped with thermal sensors (of the thermopile type) and work in the bandwidth 8 μm to 14 μm for temperature measuring in the range between −50 °C and 1000 °C. RTs of this type are routinely used and/or calibrated and certified in calibration laboratories and also used in industrial applications. They are used not only for temperature measuring but also as transfer standards (or comparators) in calibration of BB sources (of the cavity type or extended area) against reference or primary sources. The RTs we are selected are shown in Figure 5.

The main technical specifications and measurement conditions are summarized in Table 1:

The RTs were mounted into opto-mechanical mounts in front of the BB aperture, as shown in Figure 5d. BB temperature was set to 180 °C and the cooling system was adjusted to maintain the brass pieces defining apertures at 5 °C, avoiding water vapor condensation with the aid of a permanent flow of dry N_2_ on the pieces. Considering such source and aperture temperatures, the influence on the RTs of thermal radiation from the outer surface of the BB aperture is not very significant. This means that source effective diameters *d_eff_* (depending on the particular RT and aperture used in each case) can be considered as that of the aperture *d*.

**Figure 6:**
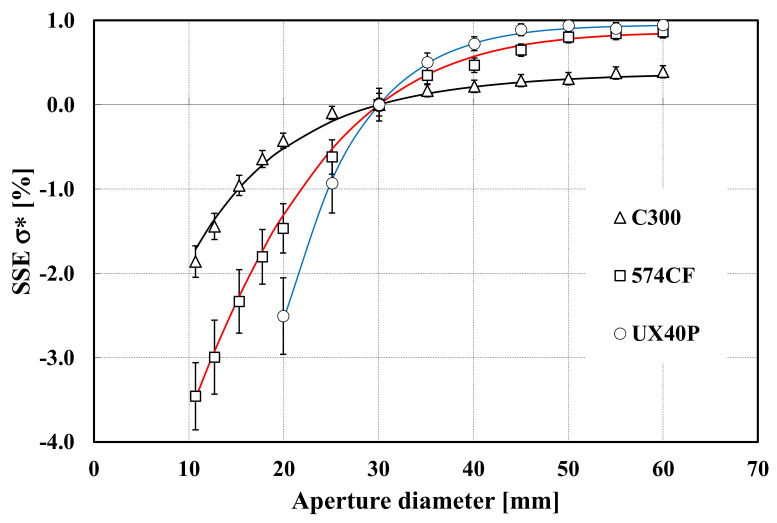
Values for σ* expressed in %, as a function of aperture diameter, for the three RTs analyzed.

**Figure 7:**
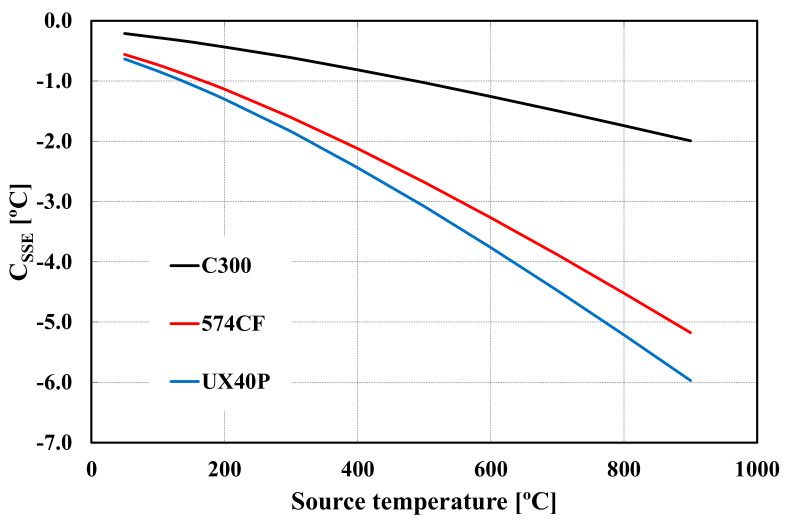
Corrections for SSE, to be applied to the three RT, as a function of temperature, between source diameters 30 mm and 50 mm.

The results for σ*, expressed as a percentage of the maximum reading (with *d*_0_ = 60 mm), are shown in Figure 6, considering calibration diameter *d_c_* = 30 mm. Figure 7 represents the correction, as a function of source temperature, to be applied to RT readings between diameter 50 mm and diameter 30 mm. In terms of uncertainty, we have assumed for *U*(*d_i_*) a value of 0.5 mm (expanded uncertainty for *k* = 2). The error bars in Figure 6, represent the expanded uncertainty *U*(σ*), obtained applying the model described above.

## 4. Discussion

The results obtained serve to illustrate the application of the procedure to various RT models. It is evident from Figure 6 and Figure 7 that SSE correction is very significant when it is compared with calibration uncertainties. Considering the three models analyzed, the uncertainties vary between 1.5 °C and 2.0 °C (expanded for *k* = 2) from 300 °C to 900 °C. As mentioned above, it is clear that SSE can be a very significant and primary source of uncertainty, either in calibration or use.

The lower uncertainty *U*(σ*) of model C300, when it compared with that of the others, is mainly due to a lower contribution by effect of diameter indetermination. As can be seen in (12), this influence factor in uncertainty strongly depends on the correction *C_SSE_*. Due to its technical characteristics, model C300 is also used in calibration laboratories (generally of the secondary type), acting principally as a comparator, for calibrating BB sources of all types against primary BB. It has better quality optics and less (a priori) imperfections, resulting in SSE. However, this cannot be taken as a general conclusion. In the course of calibration works, we have found that certain low-cost industrial RT, apparently with a low or limited metrological level, perform better in terms of SSE than others considered to be of higher quality.

**Figure 8:**
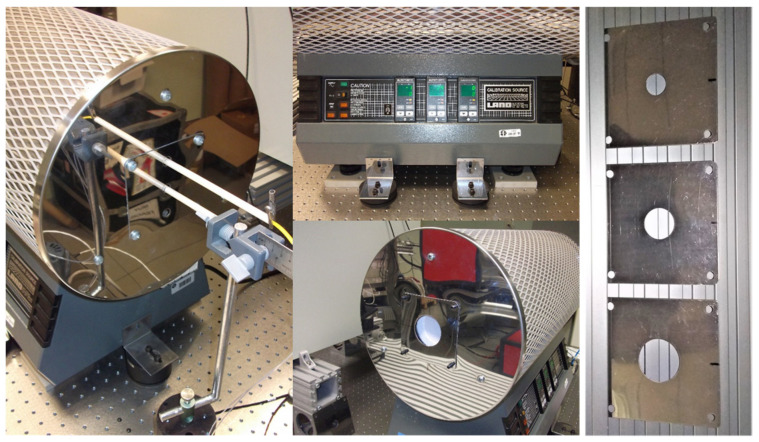
BB source and apertures plates used to validate the SSE characterization procedure.

In order to validate the procedure developed in this work, we have proposed the use of a high temperature BB source equipped with four apertures plates of diameters: 20 mm, 30 mm, 40 mm and 50 mm. Therefore, we have tried to prove if (within the uncertainty), the calculated SSE corrections are compatible with RT readings for temperatures and source different from that used to characterize this effect. To this end, we have used a BB cavity source specified for the temperature range between 150 °C and 1100 °C. The cavity is of the cylinder-conical type and is made of SiC (silicon carbide), a ceramic material with a high intrinsic emissivity. With a length of 300 mm, diameter 50 mm and apex cone angle 120°, its effective emissivity *ε_a_* was numerically calculated [18], resulting *ε_a_* = 0.998 for the bandwidth 8 μm to 14 μm. The cavity is installed in a three-zone furnace (model LAND/CARBOLITE, LandCal P1200B) equipped with two standard type R thermocouples (calibrated with expanded uncertainty *U*(*t*_90_) = 0.6 °C), for measuring cone apex temperature and longitudinal gradient. The BB source P1200B and aperture plates are shown in Figure 8.

To validate the procedure, we have used the model C300 due to its lower uncertainty. This fact allows the calculation of a more representative compatibility index (*E_n_*) for the two series of values (calculated and measured). The quantity *E_n_* measure the compatibility and is the standard figure of merit in inter-comparison exercises in metrology [19]. It is defined as:

(14) $$E_{n}=\frac{\left|v_{1}-v_{2}\right|}{\sqrt{U^{2}\left(v_{1}\right)+U^{2}\left(v_{2}\right)}}$$

where *v*_1_ and *v*_2_ are the values of the quantities to compare, *U*(*v*_1_) and *U*(*v*_2_) being the corresponding expanded uncertainties for a given coverage factor *k* (usually 2). In general, provided *E_n_* ≤ 1, the compatibility between the values is fulfilled.

The results for source temperatures 300 °C, 500 °C, 700 °C and 900 °C are shown in Table 2. For each BB temperature and each pair of apertures, the correction *C_SSE_* (8) is shown, together with its uncertainty *U_SSE_*, and the difference Δ*_RT_* between the RT readings considering both apertures, also with its uncertainty *U_RT_*. Such uncertainties were calculated considering specific influence factors, arising from the use of the high temperature BB as the uniformity and stability of the radiance temperature, display resolution of the C300 thermometer, reproducibility of the measurement procedure, etc.

With the mentioned criterion *E_n_* ≤ 1, all the measurements are compatible within the calculated uncertainty and under experimental conditions which can be reproduced in an industrial calibration laboratory framework. As stated above, the model assumes that radiation coming from the surface exterior to the apertures is negligible, which is possible when the temperature of the brass pieces is very low compared to that of the source. At 5 °C and 180 °C respectively, we found the described set-up suitable for this purpose.

### Effecive Apertures in Calibration and Comparison of RT

**Figure 9:**
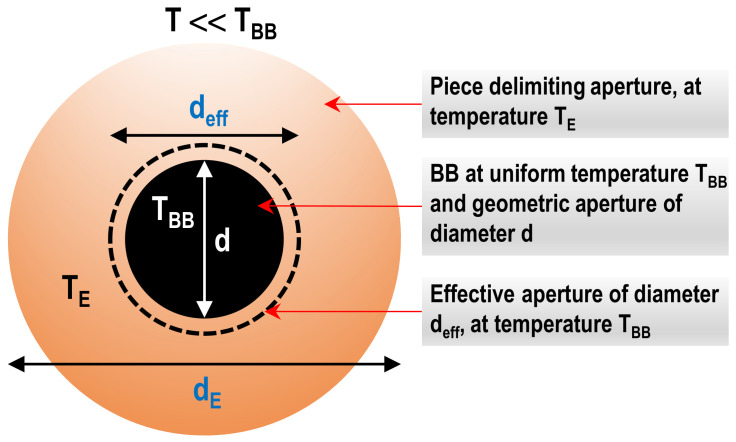
Schematic definition of a virtual or effective source, as a function of geometric aperture *d*, BB temperature *T_BB_* and external contour of diameter *d_E_* at uniform temperature *T_E_*.

As mentioned in Section 2.1, if the temperature of the piece delimiting the aperture is high, it is not possible to neglect the effect on the RT signal of radiation coming from the piece. In that case, it is common to consider a virtual source with effective diameter *d_eff_* and the same uniform temperature as the BB and producing the same signal in the RT as the real source. Figure 9 schematizes this concept.

A relatively simple setup, which can occur frequently in the RT calibration applications, is when a uniform average temperature *T_E_* can be assumed for the outer region, of diameter *d_E_*, and a temperature *T* << *T_BB_* (no thermal radiation from the external surface), as shown in the figure.

Then, *d_eff_* depends on the variables *d_E_*, *d*, *T_BB_*, *T_E_* and also on the specific characteristics of the RT in regard to its SSE corrections, evaluated as explained above.

The general model for extended and non-uniform sources, with arbitrary temperature gradients is complex [4]. However, with the previous hypothesis, it can be proved that *d_eff_* can be calculated as implicit solution of an equation that can be easily experimentally implemented, being:

(15) $$\sigma_{a,b_{1},b_{2}}^{*}\left(d_{eff}\right)=\sigma_{a,b_{1},b_{2}}^{*}\left(d\right)+\left[\frac{e^{c_{2}/\left(\lambda_{x}\cdot I_{BB}\right)}-1}{e^{c_{2}/\left(\lambda_{x}\cdot I_{E}\right)}-1}\right]\left(\sigma_{a,b_{1},b_{2}}^{*}\left(d_{E}\right)-\sigma_{a,b_{1},b_{2}}^{*}\left(d\right)\right)$$

where (for *ε_instr_* = 1) *I_BB_* is the RT indication when focused on the BB aperture center, *I_E_* is a spatial average when focused on the outer surface (assuming some temperature non-uniformity) and σ*, as we saw above, explicitly depends on diameter and parameters *a*, *b*_1_ and *b*_2_ characterizing the SSE of the particular RT. With σ* obtained under standard conditions (with a uniform BB source and no thermal radiation from the surroundings), *d_eff_* can be calculated from (15), either analytically or numerically.

One of the circumstances where correction for SSE is essential occurs in the course of inter-laboratory comparison exercises, where the accredited calibration laboratories have to periodically participate. This type of activity is essential, within the requirements of standard 17025 [3], for assuring the validity of the measurement results. In order to compare calibrations of RT performed in several laboratories, the results have to be standardized to equal source diameter. Generally, it is the diameter used by the laboratory acting as reference. This laboratory usually characterizes the RT SSE at standard conditions and calculates corrections for each temperature, as a function of the diameters provided by the participants, who rarely give effective apertures. It would be a significant improvement if the laboratories would also report the readings when the RT is focused on certain points of a well-defined region of the aperture surroundings, for example, for various diameters greater than that of the aperture. The reference laboratory would then have information to assign effective diameters to each participant, thus lowering the uncertainty corresponding to the size of source indetermination. As we have said, this influence factor strongly contributes to the uncertainty, mainly at high temperatures.

## 5. Conclusions

The procedure described has proven to be valid for its application to commercial RTs, allowing industrial calibration laboratories to have a useful tool to improve the quality and applicability of the results included in the calibration certificates they issue. It only requires having a sufficiently uniform and well characterized radiation source and a set of interchangeable elements to define variable apertures. The main advantage of this proposal is that it allows the use of low temperature BB (<200 °C). Generally, BBs of this type have larger apertures, and they are more uniform in radiance temperature than the BB designed for higher temperatures. As stated above, the results improve if the elements defining apertures can be cooled to minimize the effect of background radiation.

Making corrections to RT readings due to SSE is essential for calibrating BB when the RT is used as a comparator and the reference and calibrated sources have different aperture sizes. On the other hand, for applications where environmental conditions (or setup) in calibration, differ from that in use, the implementation of the method is more complicated or generates more uncertainty, since in general there no well-defined size of source. The common approach is to search for measurement configurations where the source can be considered infinite in size, avoiding focusing the RT on regions with strong temperature gradients.

As an example of the importance of making SSE corrections when calibration uncertainties in radiation thermometry are taking into account, we can consider an accredited calibration laboratory (say CLab1) with accredited CMC (Calibration and Measurement Capabilities) of ±2 °C (*k* = 2) at 500 °C in the waveband (8 to 14) μm. Let CLab0 the reference laboratory (higher metrological level in the traceability chain from SI) from which CLab1 obtain its metrological traceability in radiation thermometry. If CLab1 uses a RT to calibrate his 50 mm BB source, but this RT has been calibrated by CLab0 with a standard 30 mm BB, then (from Table 2) a correction of 1 °C has to be applied for a correct use of the calibration certificate given by CLab0. Then the correction cannot be considered negligible, being 50% of the CMC.

SSE in thermal imaging systems is generally insufficiently studied. Although in some aspects it is a phenomenon similar to that of RTs, there are significant differences. In addition to the effect of radiation deviating from its theoretical path due to imperfections in the optics, internal reflections, diffraction, etc., there is an essential contribution due to thermal and electrical interference between neighboring pixels [20]. The increasing use of equipment of this type in the industrial framework, due to the obvious advantage of thermal image (in addition to the temperature measurements), makes it essential that the calibration laboratories responsible for ensuring metrological traceability of the measurements have a particular methodology to evaluate SSE also in TI systems, especially that of the quantitative type.

In calibration work, TI are usually considered simply as RT with a target size calculated based on a certain selected pixel group in the focal plane array (FPA). It is necessary to study more in-depth the relationship between the classical SSE (originally defined for RT), and the specific parameters or figures of merit associated with TI, such as the point spread function (PSF) [21,22]. The problem, especially in the LWIR region, is to generate a point thermal source, avoiding thermal radiation from the region around that source. For classical qualitative TI, where the quality of thermal images is much more important than the accuracy of the temperature values shown, standardized tests are used to determine the minimum resolvable temperature difference (MRTD) based on the spatial and thermal frequency of a slit pattern. Such tests are generally considered pass/fail tests and are not used for calibration or calculation of correction factors, as required by quantitative applications. Since technological developments and industrial and scientific applications are increasingly directed towards quantitative thermography, it will be necessary to develop specific procedures that incorporate classic techniques typical of RT to the standardized procedures typical of qualitative TI.

Finally, the method described for the calculation of effective diameters, can be a useful tool for the improvement of results, either in RT calibration or in inter-comparison exercises. Although the model described is quite simple, it implies an improvement when the background temperature of the BB cannot be completely neglected.

## Figures and Tables

**Table 1:** Specific technical characteristics, determining the basic measurement conditions.

	IRCON, UX40P	LAND, C300	FLUKE, 574CF
Focus distance, mm	700 (focusable)	500 (focusable)	300 (fixed)
*ε_instr_*	1.00	1.00	1.00
*d_t_*, mm	17.5	9	6
Δ*λ*, μm	(8 a 14)	(8 a 13)	(8 a 14)
Resolution *r*, °C	0.1	0.1	0.1

**Table 2:** Results for the compatibility of the procedure for calculating SSE corrections, with the RT model C300 and the BB of Figure 8.

Apertures (Diameter), mm	*C_SSE_*, °C	*U_SSE_*, °C	Δ*_RT_*, °C	*U_RT_*, °C	*E_n_*
**300 °C**					
30 → 20	−1.0	0.2	−1.1	0.4	0.1
30 → 40	0.4	0.1	0.4		0.1
30 → 50	0.6	0.1	0.6		0.1
**500 °C**					
30 → 20	−1.8	0.3	−1.9	0.7	0.2
30 → 40	0.7	0.2	0.6		0.1
30 → 50	1.0	0.2	0.8		0.3
**700 °C**					
30 → 20	−2.5	0.4	−2.4	1.1	0.1
30 → 40	1.0	0.4	0.9		0.1
30 → 50	1.5	0.4	1.3		0.2
**900 °C**					
30 → 20	−3.4	0.6	−4.4	1.8	0.5
30 → 40	1.4	0.5	1.6		0.1
30 → 50	2.0	0.5	1.9		0.1

## Data Availability

Data access requests from academic investigators can be directed to the corresponding author.
